# Influência do Consumo de Suco de Laranja (Citrus Sinensis) na Remodelação Cardíaca de Ratos Submetidos a Infarto do Miocárdio

**DOI:** 10.36660/abc.20190397

**Published:** 2021-06-08

**Authors:** Bruna C. Oliveira, Priscila P. Santos, Amanda M. Figueiredo, Bruna P. M. Rafacho, Larissa Ishikawa, Silméia G. Zanati, Ana A. H. Fernandes, Paula S. Azevedo, Bertha F. Polegato, Leonardo A. M. Zornoff, Marcos F. Minicucci, Sergio A. R. Paiva

**Affiliations:** 1 Universidade Estadual Paulista Júlio de Mesquita Filho Faculdade de Medicina de Botucatu BotucatuSP Brasil Universidade Estadual Paulista Júlio de Mesquita Filho Campus de Botucatu - Faculdade de Medicina de Botucatu , Botucatu , SP - Brasil; 2 Instituto de Biociências BotucatuSP Brasil Instituto de Biociências Campus de Botucatu (UNESP), Botucatu , SP - Brasil; 3 Food Research Center FoRC São PauloSP Brasil Food Research Center FoRC , São Paulo , SP - Brasil

**Keywords:** Infarto do Miocárdio, Sucos de Frutas, Citrus Sinensis (laranja), Polifenóis, Remodelação Ventricular, Anti-Inflamatórios, Antioxidantes, Ratos

## Abstract

**Fundamento:**

O suco de laranja (SL) é rico em polifenóis com propriedades anti-inflamatórias e antioxidantes. Após o infarto do miocárdio (IM), mudanças complexas ocorrem na estrutura e na função cardíacas, processo conhecido como remodelação cardíaca (RC). O estresse oxidativo e a inflamação podem modular esse processo. Nossa hipótese foi a de que o consumo de SL atenua a RC após o IM.

**Objetivos:**

Avaliar a influência do SL sobre a RC após IM pela análise de variáveis funcionais, morfológicas, de estresse oxidativo, de inflação, e de metabolismo energético.

**Métodos:**

Um total de 242 ratos machos pesando entre 200 e 250g foram submetidos a um procedimento cirúrgico (ligação da artéria coronária ou cirurgia simulada). Sete dia após a cirurgia, os animais sobreviventes foram divididos para um dos quatro grupos: 1) SM, animais sham que receberam água e maltodextrina (n= 20); 2) SSL, animais sham que receberam SL (n= 20); 3) IM, animais infartados que receberam água e maltodextrina (n= 40); e 4) ISL, animais infartados que receberam SL (n = 40). A análise estatística foi realizada pelo teste de ANOVA com dois fatores com o teste de Holm-Sidak. Os resultados foram apresentados em média ± desvio padrão, e o nível de significância adotado foi de 5%.

**Resultados:**

Três meses depois, o IM levou à hipertrofia do ventrículo esquerdo (VE), com disfunção sistólica e diastólica, e aumento nos mediadores inflamatórios e de estresse oxidativo. Os animais que consumiram SL apresentaram menor atividade da glutationa peroxidase e maior expressão da heme-oxigenase-1 (HO-1).

**Conclusão:**

O SL atenuou a RC, e a HO-1 pode exercer um importante papel nesse processo.

## Introdução

O nome *polifenóis* , ou *compostos fenólicos* , refere-se a um grande grupo de moléculas encontradas em verduras, frutas, cereais, chá, café, cacau, soja, e suco de fruta. ^1^ Esses compostos têm sido estudados devido ao seu potencial efeito biológico na prevenção e tratamento de diferentes doenças. ^2 , 3^

Em revisão da literatura, Hyson mostrou que o suco da fruta, definido como suco puro ou 100% suco, reteve a maioria dos nutrientes e fitoquímicos da fruta íntegra e, portanto, pode ser importante no benefício e proteção da saúde humana. ^4^ O suco de laranja (SL) é fonte de compostos fenólicos na forma de diferentes flavonoides. O principal flavonoide de interesse é a hesperidina e sua forma hidrolisada, a hesperetina. ^5^ O interesse na pesquisa sobre as propriedades do SL aumentou devido à sua ação anti-inflamatória e antioxidante nas doenças crônicas. ^6^

Por exemplo, na lesão miocárdica, suplementos de antioxidantes podem ter efeito benéfico na remodelação cardíaca (RC). Em estudos usando modelo de infarto do miocárdio (IM), compostos bioativos presentes no alecrim, tomate, e chá verde, e antioxidantes tais como ácido ascórbico, quercetina, alfa-tocoferol, e vitamina A mostram efeito protetor contra a RC. ^1 , 3 , 7 - 10^

A doença cardíaca isquêmica, incluindo o IM, é uma causa importante de insuficiência cardíaca e morte em todo o mundo. Após o IM, mudanças complexas no ventrículo esquerdo (VE) podem causar alterações no tamanho, na massa, e na geometria do coração, e na função cardíaca. ^11 , 12^ Tais mudanças são definidas como RC e podem levar à insuficiência cardíaca e aumento na mortalidade. ^13^ Muitos fatores podem estar envolvidos na RC, incluindo estresse oxidativo, inflamação, fibrose, e apoptose. ^14 , 15^

No IM, a isquemia inicia a geração de espécies reativas de oxigênio (EROS). As EROS danificam diretamente as membranas celulares, ativam a resposta inflamatória, e levam à morte celular. Elas também podem atuar como sinais de transdução, estimulando o fator nuclear kappa B (NF-κB), o qual estimula a síntese de citocinas pró-inflamatórias. ^14 - 16^ Além disso, o sistema KEAP-1/Nrf2 (proteína 1 associada a ECH tipo Kelch/fator nuclear eritroide 2 relacionado ao fator 2) poderia ser ativado durante o estresse oxidativo celular, e exercer papel crítico na homeostase redox. Esse é um mecanismo universal que atua nos genes-alvo do Nrf2, conhecidos como elementos de resposta antioxidante. A glutationa peroxidase (GPx) e a heme-oxigenase-1 (HO-1) são exemplos de proteínas reguladas por esse sistema. ^17^

Estratégias terapêuticas para atenuar a RC após o IM têm sido muito estudadas. ^18 , 19^ Bloqueadores de aldosterona, inibidores da enzima conversora de angiotensina e betabloqueadores são algumas dessas estratégias. ^20^ Nesse contexto, os compostos bioativos de produtos naturais, com propriedades cardioprotetoras, tais como os flavonoides, podem ser importante adjuvante no tratamento de IM. Por outro lado, estudos mostram que um foco em padrões alimentares e dietéticos, e não em nutrientes ou fitoquímicos individuais, é melhor para a saúde cardiometabólica. ^21^ Assim, o objetivo deste estudo foi avaliar a influência da ingestão de SL sobre a RC após IM.

## Materiais e métodos

### Protocolo experimental

Todos os experimentos e procedimentos foram conduzidos de acordo com as diretrizes para o cuidado e o uso de animais em laboratório dos Institutos Nacionais da Saúde (NIH), e aprovados pela Comissão de Ética no Uso de Animais da Faculdade de Medicina de Botucatu, UNESP, São Paulo, Brasil (1126/2015). Foram utilizados 242 ratos Wistar machos, pesando 200 - 250 g. O IM foi induzido por ligação da artéria coronária, como descrito previamente. ^22 , 23^

Após a cirurgia, os animais foram colocados em caixas com seis animais cada. Sete dias depois, o primeiro estudo ecocardiográfico foi realizado para avaliar a eficácia do procedimento cirúrgico. ^24^ Com base nesse ecocardiograma, os animais foram alocados aleatoriamente em caixas com dois animais cada, para receberem SL ou uma solução de maltodextrina (M). Os grupos foram: 1) SM, animais sham que receberam solução de M (n=20); 2) SSL, animais sham que receberam SL (n=20); 3) IM, animais com infarto que receberam solução de M; e 4) ISL, animais com infarto que receberam SL (n=40). O tamanho da amostra utilizada baseou-se em outros estudos realizados em nosso laboratório. ^3 , 8 , 25^ O número de ratos no grupo infartado foi maior, uma vez que a mortalidade esperada para esses animais durante o período experimental é de aproximadamente 50%. Além disso, somente animais com área infartada do VE maior que 30% foram incluídos no estudo. ^24^

**Figura 1:**
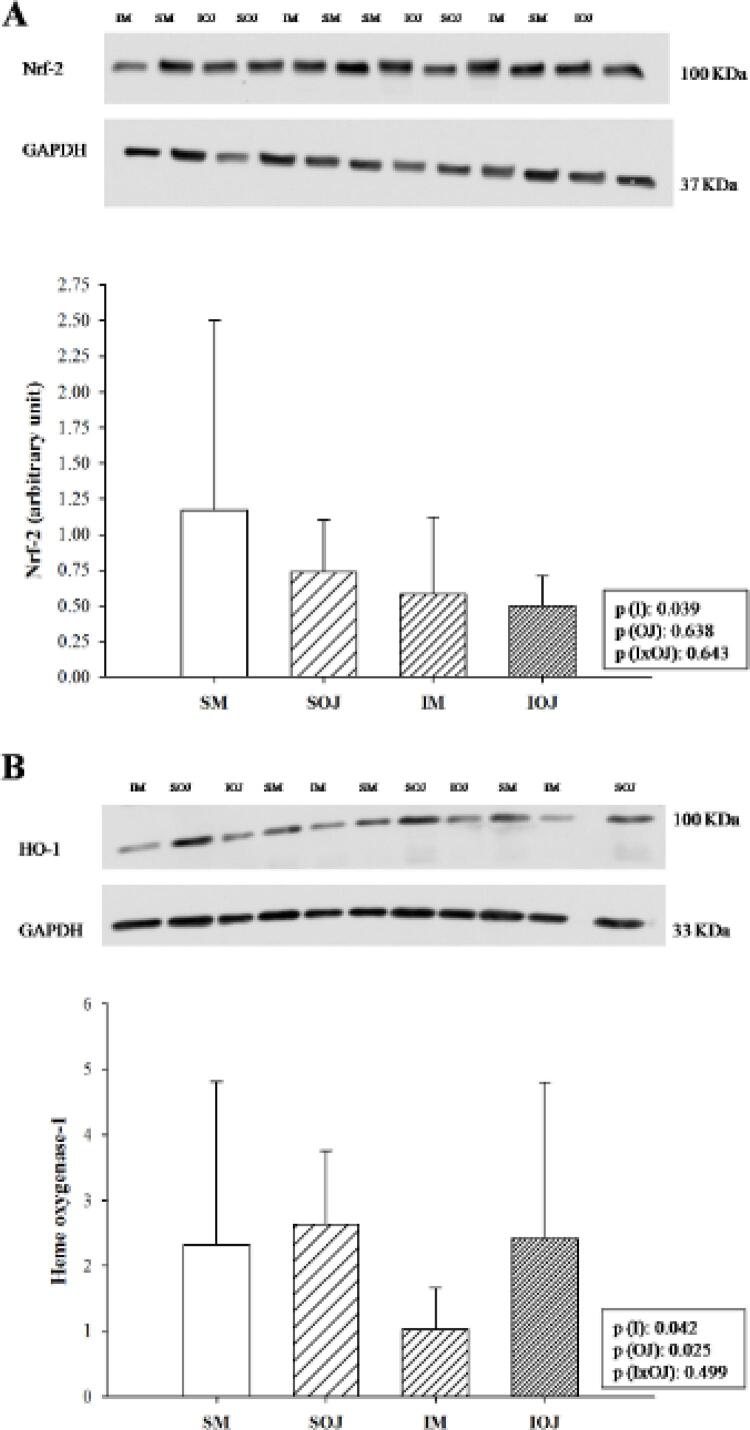
– Expressão de fator nuclear eritroide 2 relacionado ao fator 2 (Nrf2) e heme-oxigenase-1 (OH-1) em ratos sham e ratos infartados por Western blot. Gráfico de barras mostrando a expressão de Nrf-2 e de HO-1 em cada grupo (A) expressão do Nrf-2 e Western blot representativo; tamanho da amostra: 8 animais em cada grupo; (B) expressão de HO-1 e Western blot representativo; tamanho da amostra: SM = 5; SSL = 6; IM = 5; ISL = 5; GAPDH gliceraldeído -3-fosfato-desidrogenase; dados expressos em média ± DP. p(I): valor p entre animais infartados e não infartados; p(SL): valor p entre animais que receberam maltodextrina e animais que receberam suco de laranja; p(IxSL): representa o valor de p quando houve interação entre fatores relacionados a infarto e fatores relacionados ao consumo de suco de laranja

O alimento era oferecido à vontade ( *ad libitum* ). Os animais foram tratados durante três meses, e a mortalidade foi avaliada nesse período ( Figura 1 dos dados suplementares). Os ratos foram acondicionados em temperatura controlada (22 ± 2°C), com um ciclo claro-escuro de 12 horas.

### Ligação da artéria coronária

O IM foi induzido por ligação da artéria coronária, conforme descrito previamente. ^22 , 23^ Em resumo, os ratos foram anestesiados com cetamina (70mg/Kg) e xilazina (1mg/Kg). Após toracotomia esquerda, o coração foi retirado. O átrio esquerdo foi retraído para facilitar a ligação da artéria coronária esquerda, com fio mononylon 5.0 entre a saída da artéria pulmonar e o átrio esquerdo. O coração foi recolocado no tórax, os pulmões inflados com pressão positiva, seguido por fechamento da toracotomia. Também se criou um grupo sham, em que os animais foram submetidos à cirurgia sem oclusão coronária. Após o efeito da anestesia, os ratos foram medicados oralmente com Metamizol sódico (30 mg / kg Dipirona®, Biovet, Vargem Grande Paulista, São Paulo, Brasil).

### Suco de laranja

Os grupos SSL e ISL receberam suco de laranja em regime *ad libitum* . Os grupos controles (SM e IM) receberam uma solução de água e M na concentração de 100g/L. A solução de M foi dada aos animais controle para fornecer a mesma quantidade de carboidratos que o SL. O tratamento foi iniciado sete dias após a cirurgia. O SL e as soluções de M eram trocadas a cada 24 horas, e o consumo foi monitorado diariamente. A composição nutricional do SL é apresentada nos dados suplementares.

### Estudo ecocardiográfico

Após três meses, todos os ratos foram pesados e avaliados por ecocardiografia transtorácica. ^26 , 27^ Para o estudo ecocardiográfico, os ratos foram anestesiados com injeção intramuscular de solução de cetamina (50 mg/kg) e xilazina (1 mg/kg). As medidas foram feitas pelo mesmo observador, seguindo o método de última geração recomendado pela Sociedade Americana de Ecocardiografia e a Associação Europeia de Ecocardiografia. ^28^ O ecocardiograma foi realizado com o sistema General Electric Vivid S6 System (GE Medical Systems, Tirat Carmel, Israel), com sonda *phased array* de 5 a 12-MHz.

Após o ecocardiograma, os animais foram eutanasiados com uma alta dose de pentobarbital, e os corações foram removidos. O VE foi isolado e amostras foram retiradas e imediatamente congeladas e armazenadas a -80 ^o^ C. Um corte transversal do VE foi separado e fixado com formalina tamponada 10%, e embebidos em parafina para estudo histológico.

### Análise morfométrica

Cortes de cinco micrômetros de espessura foram marcados com hematoxilina e eosina para cálculo do tamanho do infarto conforme descrito anteriormente. Todos os animais foram incluídos na análise morfométrica. Após o cálculo do tamanho do infarto, os animais infartados com menos de 30% do VE de área infartada foram excluídos das análises. Todas as imagens foram coletadas com câmera de vídeo acoplada ao microscópio (Leica); as imagens foram analisadas usando o software Image-Pro Plus 3.0 (Media Cybernetics, Silver Spring, MD).

### Hidroperóxido lipídico no tecido cardíaco, atividade de enzima antioxidante, e metabolismo energético cardíaco

Amostras do VE (100mg) foram usadas para medidas de proteína total e hidroperóxido lipídico (HL), e atividade das seguintes enzimas antioxidantes – GPx (E.C.1.11.1.9), superóxido dismutase (SOD, E.C.1.15.1.1), e catalase (E.C.1.11.1.6). O metabolismo energético cardíaco foi avaliado pela atividade da 3-hidroxiacil coenzima-A desidrogenase (OHADH; E.C.1.1.1.35.), fosfofrutoquinase (PFK; E.C.2.7.1.11), lactato desidrogenase (LDH; E.C.1.1.1.27), piruvato desidrogenase (E.C.1.2.4.1), citrato sintase (CS; E.C.4.1.3.7.), e trifosfato de adenosina (ATP) sintase (EC 3.6.3.14). ^3 , 9^ Os testes de atividade enzimática foram realizados a 25 ^o^ C com um leitor de microplaca (µQuant-MQX 200-EONC com o software Gen5 2.0 conectado a um sistema de controle; Bio-Tec Instruments, VT, EUA). Todos os reagentes foram obtidos de Sigma (Sigma-Aldrich, St. Louis, MO, USA).

### Mediadores inflamatórios

Concentrações de interferon-γ (IFN-γ) e interleucina-10 (IL-10) nas amostras do VE foram determinadas por ELISA seguindo-se as instruções do fabricante (R&D Systems, Minneapolis, MN).

### Western blot

O teste de Western blot foi realizado para analisar a expressão proteica da GPx-1 (ab 22604 - Abcam Inc, Cambridge), HO-1 (ab13248 - Abcam Inc, Cambridge), NF-kB total e fosforilada (NF-kB- sc 8008 e sc 3302- Santa Cruz Biotechnology, Inc, Europa), e sirtuína-1 (Sirt-1- sc 15404-Santa Cruz Biotechnology, Inc, Europa), no extrato celular total. Para determinar o fator nuclear eritroide 2 (Nrf-2-sc 722-Santa Cruz Biotechnology Inc, Europe), amostras do VE foram extraídas utilizando-se tampão de extração nuclear. ^9^ As amostras foram separadas em gel de poliacrilamida-dodecil sulfato de sódio (SDS-PAGE) a 10%, e as proteínas transferidas para uma membrana de nitrocelulose. A membrana foi bloqueada com leite em pós desnatado (5%) e em seguida incubada com anticorpo primário e anticorpo secundário. Gliceraldeído-3-fosfato desidrogenase (GAPDH) (sc 32233, Santa Cruz Biotechnology, Inc., Europa) foi usada para normalização das proteínas.

### Análise estatística

A normalidade dos dados foi verificada pelo teste de Kolmogorov–Smirnov. Os dados foram apresentados como média ± desvio padrão (DP). As variáveis com distribuição normal foram analisadas pelo teste de variância com dois fatores, que fornece três valores p: 1) fator 1, presença de IM (I); 2) fator 2, ingestão de SL (SL); e 3) interação entres os fatores I e SL. Na análise de variância com dois fatores, assume-se a normalidade da distribuição dos dados. Se uma variável não se ajusta à distribuição normal, realiza-se transformação dos dados. O teste t de Student não pareado foi usado para análise do ecocardiograma inicial. O teste do qui-quadrado foi usado para avaliar mortalidade, e o teste t de Student não pareado foi usado para avaliar o tamanho do infarto nos animais infartados. As diferenças foram consideradas estatisticamente significativas se o valor de p fosse inferior a 0,05. As análises estatísticas foram realizadas usando o programa SigmaPlot para Windows 12.0 (Systat Software Inc., San Jose, CA).

## Resultados

O ecocardiograma inicial mostrou que os animais dos dois grupos de animais infartados não apresentaram diferenças na área sistólica e diastólica ou no tamanho do infarto ( Tabela 1 do material suplementar).

**Tabela 1:** – Tamanho do infarto, ecocardiograma final, e análise morfométrica

Variável	SM (n = 19)	SSL (n = 20)	IM (n = 9)	ISL (n = 9)	p (I)	p (OJ)	p (I×OJ)
Peso corporal (g)	454±47,9	480±56,3	443±66,6	462±25,9	0,338	0,135	0,852
FC (bpm)	290±30,9	296±34,9	268±26,7	324±30,0 ^aB^	0,756	0,001	0,009
AE/Ao	1,32±0,09	1,21±0,09 ^b^	1,78±0,21 ^A^	1,42±0,18 ^Ba^	<0,001	<0,001	0,003
DDVE/BW (mm/kg)	15,9±1,31	15,0±1,79	22,1±2,70	20,2±2,10	<0,001	0,019	0,398
DSVE/BW (mm/kg)	6,67±0,87	6,02±0,89	15,1±2,70	12,8±2,70	<0,001	0,007	0,556
IMVE (g/kg)	1,63±0,22	1,56±0,27	2,62±0,60	2,36±0,36	<0,001	0,124	0,640
FAC (%)	67,3±8,28	67,4±8,27	34,5±8,28	36,6±8,28	<0,001	0,661	0,707
Onda E (ms)	79,7±11,3	81,4±7,16	89,1±10,8	80,7±19,2	0,204	0,325	0,139
Onda A (ms)	49,9±8,28	52,6±7,16	42,5±16,5	62,7±24,9 ^a^	0,730	0,004	0,025
S´ média (cm/s)	5,78±0,04	5,82±0,31	4,60±0,30	5,01±0,3	<0,001	0,028	0,078
E´ média (cm/s)	5,62±0,44	5,82±0,45	4,23±0,60	4,77±0,60	<0,001	0,054	0,357
A´ média (cm/s)	3,67±0,44	4,02±0,45	4,54±0,90	5,55±1,20	0,058	0,058	0,28
Razão E/E´	13,7±3,49	14,1±1,79	21,3±3,30 ^A^	16,9±2,40 ^Ba^	<0,001	0,002	0,003
Peso VE/peso corporal (mg/g)	1,85±0,13	1,93±0,27	2,13±0,42	2,10±0,15	0,005	0,550	0,822
Peso VD/peso corporal (mg/g)	0,46±0,09	0,43±0,05	0,65±0,24	0,59±0,27	<0,001	0,294	0,776

*Dados expressos em média ± desvio padrão. n: número de animais incluídos em cada grupo; SM: animais sham que receberam maltodextrina; SSL: animais sham que receberam suco de laranja; IM: animais infartados que receberam maltodextrina; ISL: animais infartados que receberam suco de laranja; FC: frequência cardíaca; AE: diâmetro do átrio esquerdo; Ao: diâmetro da aorta; DDVE: diâmetro diastólico final do ventrículo esquerdo; DSVE: diâmetro sistólico final do ventrículo esquerdo; IMVE: índice da massa do ventrículo esquerdo (massa do ventrículo esquerdo/peso corporal); FAC: fractional area change (variação da área do ventrículo esquerdo); onda E: velocidade de movimentação do anel mitral no início da diástole; onda A: velocidade de movimentação do anel mitral no final da diástole; média S´: velocidade média de movimentação do anel mitral septal e lateral; A´ e E´ médias: velocidade média de movimentação do anel mitral septal e lateral na diástole (E’: início e A’: final); VE: ventrículo esquerdo/ VD: ventrículo direito; pI:valor p do efeito do infarto; pSL: valor**p**do efeito do consumo de suco de laranja. pIxSL: valor p da interação.Números em negritorepresentam efeitos estatisticamente significativos.* ^*a*^ *: IM≠ISL;* ^*b*^ *: SM≠SSL;* ^*A*^ *: SM≠IM e* ^*B:*^ *SSL≠ISL.*

Durante os três meses de experimento, a mortalidade foi de 5% no grupo SM (um rato morreu), 0% no grupo SSL, 22,5% no grupo IM (9 ratos morreram), e de 22,5% no grupo ISL (9 ratos morreram). Quando todos os grupos foram analisados, observou-se uma diferença na mortalidade entre os grupos (p=0,04). Contudo, a mortalidade não foi diferente entre os grupos infartados (p=0,836). Após o período de consumo de SL, realizou-se a eutanásia dos animais sobreviventes. Em seguida, efetuou-se a análise histológica do VE dos animais infartados para verificar o tamanho do infarto ( Figura 1 do material suplementar) (IM= 40,1±7,41%; ISL=38,1±5,76%; p= 0,528). O peso corporal final não foi diferente entre os grupos ( Tabela 1 ).

### Efeito do IM nos corações dos ratos

O IM levou à RC. Quanto aos dados morfológicos, o IM causou aumento no diâmetro diastólico do VE/peso corporal final, diâmetro sistólico do VE /peso corporal final, diâmetro do átrio esquerdo/aorta, índice de massa do ventrículo esquerdo (IMVE), peso do VE/peso corporal final Tabela 1 ), e espessura da parede posterior do VE/peso corporal final, espessura da parede do septo interventricular/peso corporal final, e diâmetro atrial esquerdo/peso corporal final ( Tabela 1 do material suplementar). Essas mudanças caracterizam o aumento das cavidades esquerdas e hipertrofia do VE. O IM afetou a função cardíaca sistólica, conforme os valores mais baixos da variação de área do VE ( *fractional area change* , FAC) e S’ média ( Tabela 1 ), encurtamento endocárdico, e fração de ejeção ( Tabela 2 do material suplementar). A função diastólica também foi afetada, indicado por uma redução na E’ média ( Tabela 1 ), tempo de desaceleração da onda E, E’ lateral, e E’ septal ( Tabela 2 do material suplementar), e aumento na onda A, média da A’, razão E/E’ ( Tabela 1 ), índice de Tei, razão E/A, tempo de relaxamento isovolumétrico ajustado pela frequência cardíaca, A’ lateral e A’ septal.

**Tabela 2:** – Marcadores de estresse oxidativo e marcadores inflamatórios

Variável	SM (n = 8)	SSL (n = 8)	IM (n = 4)	ISL (n = 4)	p (I)	p (OJ)	p (I×OJ)
LH (nmol/g)	209±28,3	215±28,3	256±28,0	277±26,0	<0,001	0,293	0,573
Catalase (µmol/g)	64,4±6,79	61,8±7,07	55,4±7,00	58,1±7,60	0,056	0,993	0,404
SOD (nmol/mg)	12,9±2,83	13,5±2,83	18,7±1,80	17,1±1,40	<0,001	0,639	0,355
Atividade da GSH-px (nmol/mg)	62,5±6,51	50,2±7,07	58,0±7,00	45,3±6,00	0,134	<0,001	0,951
Expressão da GSH-px (unidade arbitrária)	7,56±7,39	7,62±10,8	6,63±5,82	6,37±3,78	0,600	0,649	0,546
IL-10 (pg/mg)	23,9±7,92	39,6±13,0 ^b^	42,0±9,60 ^A^	34,1±7,60	0,135	0,352	0,008
IFN-γ (pg/mg)	7,1±2,26	13,2±3,96 ^b^	17,7±6,80 ^A^	11,2±2,00 ^a^	0,037	0,928	0,003
NF-κB (unidade arbitrária)	7,03±11,0	4,61±4,98	3,40±3,12	6,80±4,00	0,942	0,330	0,266
p NF-κB (unidade arbitrária)	5,40±10,2	3,18±2,40	3,28±3,12	5,22±3,34	0,587	0,084	0,980
Sirt 1 (unidade arbitrária)	1,81±0,82	2,34±1,41	2,42±1,44	1,65±0,56	0,925	0,813	0,203

*Dados expressos em média ± desvio padrão. n: número de animais incluídos em cada grupo; SM: animais sham que receberam maltodextrina; SSL: animais sham que receberam suco de laranja; IM: animais infartados que receberam maltodextrina; ISL: animais infartados que receberam suco de laranja; HL: hidroperóxido lipídico; SOD: superóxido dismutase; GSH-px: glutationa peroxidase; IL-10: interleucina-10; INF-γ: interferon- γ; NF-κB: fator nuclear κB total; e p NF-κB: fator nuclear κB fosforilado; Sirt 1: sirtuína 1. pI:valor p do efeito do infarto; pSL: valor p do efeito do consumo de suco de laranja. pIxSL: valor p da interação. Números em negrito representam efeitos estatisticamente significativos. ^a^: IM≠ISL; ^b^: SM≠SSL; ^A^: SM≠IM e ^B:^ SSL≠ISL.*

**Figura 2:**
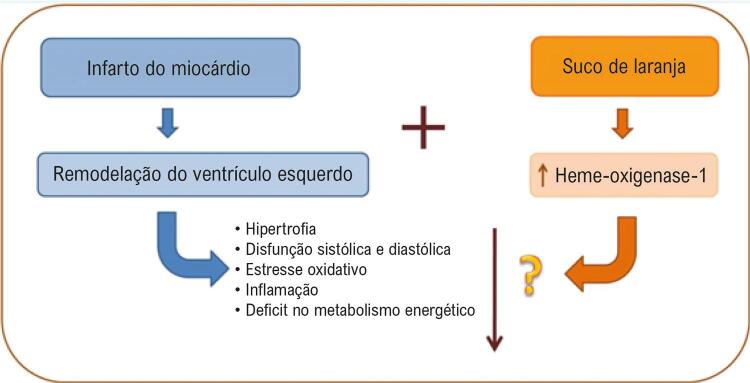
– Esquema ilustrativo dos principais achados do estudo.

O IM também aumentou o estresse oxidativo, demonstrado pelo aumento na atividade de LH e SOD ( Tabela 2 ), menor expressão da HO-1 (Figura 1A), e ocorreu a menor expressão do Nrf-2 (Figura 1B). Os mediadores inflamatórios IL-10 e INF-γ foram mais altos no IM ( Tabela 2 ), e não houve diferença nos níveis de NF-κB ou Sirt-1 entre os animais infartados e não infartados ( Tabela 2 e Figura 2 do material suplementar). Observou-se maior oxidação de carboidratos que de ácidos graxos, e menor metabolismo energético, demonstrados por maior atividade de LDH e PFK, e menor atividade de OHADH, CS, e ATP sintase. Não foi observada diferença para a atividade do piruvato desidrogenase ( Tabela 3 ).

**Tabela 3:** – Enzimas envolvidas no metabolismo energético cardíaco

Variável	SM (n = 8)	SSL (n = 8)	IM (n = 4)	ISL (n = 4)	p (I)	p (SL)	p (I×SL)
PFK (nmol/g)	128±17,0	112±24,6	139±26,0	178±26,0 ^aB^	<0,001	0,257	0,011
LDH (nmol/mg)	88,5±18,7	82,6±18,1	111±18,6	134±16,	<0,001	0,320	0,092
PDH (nmol/g)	344±53,7	385±31,1	345±100	341±42,0	0,386	0,461	0,376
OHADH (nmol/mg)	33,2±6,22	33,9±6,22	23,8±8,40	24,5±2,80	0,003	0,798	0,992
CS (nmol/mg)	50,0±6,22	49,4±6,51	34,5±7,40	40,5±4,80	<0,001	0,350	0,254
ATP sintase (nmol/mg)	21,0±3,11	27,8±5,37	11,4±1,20	15,9±4,40	<0,001	0,005	0,532

*Dados expressos em média ± desvio padrão. n: número de animais incluídos em cada grupo; SM: animais sham que receberam maltodextrina; SSL: animais sham que receberam suco de laranja; IM: animais infartados que receberam maltodextrina; ISL: animais infartados que receberam suco de laranja; PFK: fosfofrutoquinase; LDH: lactato desidrogenase; PDH: piruvato desidrogenase; OHADH: 3-hidroxiacil coenzima-A desidrogenase; CS: citrato sintase; ATP: adenosina trifosfato. pI: valor p do efeito do infarto; pSL: valor p do efeito do consumo de suco de laranja. pIxSL: valor p da interação. Números em negrito representam efeitos estatisticamente significativos. ^a^: IM≠ISL; ^b^: SM≠SSL; ^A^: SM≠IM e ^B:^ SSL≠ISL.*

### Efeito do consumo de SL sobre o coração

A ingestão de SL causou redução da cavidade do VE, com valores mais baixos do diâmetro diastólico final do VE (DDVE) e diâmetro sistólico final do VE (DSVE); melhorou a função sistólica, com valores mais altos de S’ médio; e melhorou a função diastólica, com menor diâmetro do átrio esquerdo ajustado para o diâmetro da aorta ( Tabela 1 ) ^29^ após o IM. Não foram observadas diferenças para outras variáveis ecocardiográficas ( Tabela 1 ). As outras variáveis relativas à função não foram valorizadas devido à elevada frequência cardíaca ^30^ no grupo SL ( Tabela 2 do material suplementar).

Além disso, os animais que consumiram SL apresentaram atividade mais baixa da GSH-Px ( Tabela 2 ). Não foram observadas diferenças para atividade de LH, SOD, catalase, ou expressão de GSH-Px ( Tabela 2 e Figura 2 do material suplementar).

Quanto aos mediadores inflamatórios, o grupo SSL apresentou IL-10 e INF-γ mais elevados em comparação ao grupo SM ( Tabela 2 ). O grupo ISL apresentou valores de INF-γ mais baixos que o grupo IM ( Tabela 2 ). Não houve diferenças para NF-κB ou Sirt-1 entre os animais que consumiram e os que não consumiram SL ( Tabela 2 e Figura 2 do material suplementar).

Observou-se melhora no uso de substratos nos animais que consumiram SL. Observamos valores mais altos para atividade da PFK no grupo ISL em comparação ao grupo IM, e atividade mais alta da ATP sintase nos animais que consumiram SL. Não se observou diferenças na atividade de outras enzimas do metabolismo energético entre os grupos ( Tabela 3 ).

Um resultado interessante que os animais que consumiram SL apresentaram maior expressão de HO-1 (Figura 1A), apesar de não terem apresentado diferença na expressão de Nrf2 (Figura 1B).

## Discussão

No presente estudo, IM induzido por ligação da artéria coronária em ratos resultou em hipertrofia do VE e disfunção diastólica, o que foi compatível com alterações observadas no infarto crônico. ^11^ Nossos dados também mostraram aumento no estresse oxidativo e marcadores inflamatórios, bem como alterações no metabolismo energético, com deficiência na β-oxidação de ácidos graxos. Essas alterações caracterizam o processo de RC. ^31 - 33^ Também observamos redução na expressão de Nrf-2 e HO-1. Na fase crônica do IM, a via do Nrf2 pode estar reduzida por expressão anormal do gene-alvo do Nrf2, afetando a manutenção da homeostase redox via enzimas medidas por elementos de resposta antioxidante. ^34^ Em estudo prévio conduzido em nosso laboratório com modelo de IM, observamos expressão mais baixa de Nrf2 e HO-1. ^3^ Esses achados sugerem ou uma menor expressão ou um maior catabolismo da proteína Nrf2, levando, assim, à menor síntese de HO-1.

No presente estudo, o consumo de SL resultou na atenuação da RC nos animais infartados. Essa atenuação pode ser observada na diminuição da cavidade do VE (DDVE e DSVE), e na melhora da função sistólica, caracterizada pelo aumento no S’ médio, ^35^ e na função diastólica (menor diâmetro do átrio esquerdo). No estudo de Yu et al., ^36^ os ratos infartados por ligação da artéria coronária esquerda, tratados com hesperidina por quatro semanas, apresentaram DDVE e DSVE mais baixos e melhor função sistólica que os animais infartados. Esses dados são similares aos nossos, e podem indicar o efeito da hesperidina do SL sobre o processo de RC. Em outro estudo, outro composto fenólico, a hesperetina, também apresentou efeito sobre o coração. Em um modelo de sobrecarga de pressão, Deng et al., ^37^ encontraram valores mais baixos de DDVE e de DSVE oito semanas após a administração de hesperetina.

O IM leva a desequilíbrio entre a produção de EROS e defesas antioxidantes, levando a estresse oxidativo. Após isquemia, algumas EROS danificam membranas celulares, iniciando o processo de peroxidação lipídica. ^38^ Por exemplo, Bagatini et al., ^39^ descreveram aumento na peroxidação lipídica em pacientes com IM. Nossos resultados também mostraram maior concentração de hidroperóxidos lipídicos nos animais infartados em comparação aos animais não infartados. ^40^ A enzima SOD é a primeira defesa do organismo contra EROS. Nosso estudo mostrou que os animais infartados, em comparação aos não infartados, apresentaram maior atividade da enzima SOD, conforme descrito anteriormente. ^3 , 25^ Em relação ao consumo de SL, observamos que os animais que consumiram SL apresentaram menor atividade da GSH-Px. Resultado semelhante foi apresentado por Selvaraj e Pugalendi ^41^ no modelo de isquemia induzida por isoproterenol: os ratos que receberam hesperidina apresentaram atividade mais baixa das enzimas antioxidantes, entre eles, a GSH-Px. ^41^

Em relação ao metabolismo energético, o coração, assim como outros órgãos, consegue adaptar-se e utilizar o melhor substrato energético em cada situação. A PFK atua na regulação da glicólise, e catalisa a fosforilação de glicose em frutose-6-fosfato e subsequentemente em frutose 1,6-bisfosfato. ^42^ A PFK é ativada quando as concentrações de ATP são reduzidas e é inibida quando as células têm reserva suficiente de ATP e de outros substratos, tais como ácidos graxos. ^42^ Nossos dados mostraram valores mais altos de PFK em animais infartados com ingestão de SL. Esses dados mostram que atividade aumentada da PFK pode levar à regulação da via glicolítica, fornecendo mais substrato para produção de energia. Outro achado importante que indica maior uso de substrato é a maior atividade da ATP sintase em animais que receberam SL.

Além do estresse oxidativo e alterações metabólicas, observamos que os animais infartados que receberam SL apresentaram valores mais baixos de IFN-γ. Uma vez que a fase crônica da inflamação está relacionada a uma produção aumentada de IFN-γ, ^43^ nossos resultados sugerem uma fase mais avançada em direção à resolução do processo inflamatório. Um resultado interessante é que animais sham que consumiram SL mostraram efeito imunomodulatório, indicado pelos valores mais altos de IL-10 e INF-γ. Similar aos nossos achados em animais do grupo sham, os quais não sofreram nenhuma lesão cardíaca, estudos com humanos sadios, de meia idade, relataram que o SL alterou a expressão gênica em leucócitos para um perfil anti-inflamatório e antiaterogênico, ^44^ e promoveu uma proteção precoce de células sanguíneas mononucleares contra dano oxidativo no DNA. ^45^ Além disso, o consumo de SL com a refeição rica em carboidratos preveniu o estresse oxidativo e inflamatório induzido pela refeição. ^46^

Outro achado interessante em nosso estudo foi os valores mais elevados de HO-1 em animais que consumiram SL. Lin et al., ^47^ em 2005, também mostraram que a hesperetina induziu a expressão proteica de HO-1. ^47^ A enzima HO-1 exerce ação importante na homeostase celular devido à sua ação catabólica no grupo heme das hemoproteínas, gerando subprodutos tais como ferro, biliverdina e monóxido de carbono. Por meio desses subprodutos, a HO-1 exerce ação inflamatória antioxidante, e antiapoptótica. ^48 , 49^ Além dessa função clássica, a HO-1 participa na sinalização celular amplificando a ação dos indutores (heme, oxidantes, citocinas, forças hemodinâmicas, fatores de crescimento, hipóxia, e hormônios) de fatores de transcrição. ^48^

Wang et al., ^50^ mostraram que a HO-1 é importante para a homeostase cardíaca, protegendo o órgão contra isquemia e lesões induzidas pela reperfusão, e danos oxidativos. ^50^ Em outro estudo, a administração de hemina em camundongos infartados induziu a ativação de HO-1, o que causou uma mudança nos macrófagos para um fenótipo anti-inflamatório (M2), redução da expansão da cicatriz do infarto, e melhora da função cardíaca. ^51^ Assim, HO-1 aumentada também pode exercer importante papel na atenuação da RC pelo SL ( Figura 2 ). Ainda, esse aumento foi independente da via do Nrf2, uma vez que o SL não causou alterações na expressão dessa proteína. Similar aos nossos achados, Wang et al., ^52^ encontraram que a isoliquiritina e a isoliquiritigenina, flavonoides derivados do alcaçuz, induziram a expressão de HO-1 independentemente da expressão de Nrf2. ^52^ A expressão de HO-1 pode ser induzida por diferentes vias e variar de acordo com o modelo e tratamento utilizado. ^47^

### Limitações

O SL usado no estudo foi um suco comercial, pronto para consumo, pasteurizado, livre de conservantes e açúcar. A escolha por esse suco foi para garantir a padronização. No entanto, é possível que o uso de outros tipos de sucos, elaborados com outros tipos de laranjas, poderia levar a respostas diferentes.

## Conclusão

O SL atenuado por RC após IM, com diminuição do diâmetro do VE e melhora da função sistólica e diastólica; HO-1 pode desempenhar um papel importante neste processo.

## * Material suplementar

Para informação adicional, por favor, clique aqui.
