# Supplementary material for: Influência do Consumo de Suco de Laranja (Citrus Sinensis) na Remodelação Cardíaca de Ratos Submetidos a Infarto do Miocárdio
Source: Arq Bras Cardiol. 2021 Jun 8;116(6):1127–36. [Article in Portuguese] doi: 10.36660/abc.20190397 (PMC8288524; doi:10.36660/abc.20190397)
Supplement: Supplementary file 1 [file 2019-0397-material-suplementar.pdf]

Supplemental file

Nutrition information and compound determination of orange juice.

**Portion: 200 mL (1 cup)**		% DV*
kcal = kJ		
Energetic value	86 kcal = 365 kJ	4.3
Carbohydrate	20 g	6.7
Protein	1.5 g	2.0
Total fat	0 g	0
aturated fat	0 g	0
Trans fats	0 g	
Food fiber	0 g	0
Sodium	0 g	0
Calcium	20 mg	2.0
Magnesium	9 mg	1.4
Phosphor	24 mg	
Potassium	174 mg	
Iron	0.2 mg	1.4
Copper	0.04 mg	
Vitamin C	64 mg	142
Folic acid	58 mg	14
Total phenolic compounds	85.8±1.2[a] mg/100 mL	
Antioxidant activity	7.14±0.29[a] DPPH/L	
Ascorbic acid	60.1±1.80[a] mg/100 mL	
Total carotenoids expressed as beta carotene	136±8.0 [a] µg/100 mL	

* Daily reference values based on 2000 kcal or 8400 kJ diet.

[a] Values presented as mean ± SD

Table 1. Initial echocardiographic study.

**Variable**	**IM (n = 40)**	**IOJ (n = 40)**	***P***
Systolic area (mm^2)	38.7±14.3	36.3±13.5	0.446
Diastolic area (mm^2)	58.7±12.7	55.3±12.2	0.225
Initial infarction size (%)	40.8±7.40	44.8±12.5	0.085

Data are expressed as mean ± standard deviation. I: infarcted animals; M: animals that received maltodextrin; OJ: animals that received orange juice. *p*: value of Student's *t*-test.

Table 2. Final echocardiogram analysis.

Variable	SM (n = 19)	SOJ (n = 20)	IM (n = 9)	IOJ (n = 9)	*P* (I)	*P* (OJ)	*P* (I×OJ)
PWT/BW (mm/kg)	3.46±0.39	3.38±0.45	3.75±0.30	3.78±0.30	**0.007**	0.832	0.661
ISWT/BW (mm/kg)	3.64±0.39	3.52±0.45	3.01±0.30	2.98±0.30	**<0.001**	0.557	0.767
LA/BW (mm/kg)	10.9±1.31	9.71±1.34	14.7±1.50	11.7±1.50	**<0.001**	**<0.001**	0.05
HR (bpm)	290±30.9	296±34.9[B]	268±26.7[a]	324±3.00[aB]	0.756	0.001	**0.009**
FS (%)	58.1±2.62	59.9±4.02	31.8±5.40	36.8±9.00	**<0.004**	0.129	0.181
EF (%)	92.5±1.31	93.4±1.79	67.8±7.50	73.4±10.8	**<0.001**	0.179	0.219
Tei index	0.51±0.13	0.45±0.13	0.75±0.12	0.71±0.12	**<0.001**	0.184	0.891
E/A	1.62±0.26	1.56±0.18	2.58±1.20	1.81±1.50	**<0.001**	0.456	0.310
EDT (m/s)	47.0±4.79	46.8±4.03	41.6±10.8[a]	43.1±10.5[a]	**0.028**	0.759	0.684
$IRT/RR^{0.5}$ (ms)	47.0±7.41	47.0±4.47[B]	55.6±18.9[a]	75.7±16.5[aB]	<0.001	<0.002	**0.003**
S septal (cm/s)	5.68±0.39[A]	5.79±0.40[B]	4.10±0.30[Aa]	4.76±0.30[aB]	<0.001	0.002	**0.022**
S lateral (cm/s)[#]	5.87±0.44	5.86±0.40	5.11±0.30	5.25±0.30	**<0.001**	0.953	0.459
A’ septal (cm/s)	3.90±0.44	4.27±0.44	4.85±1.20	6.47±2.10	**<0.001**	**0.003**	0.057
A’ lateral (cm/s)	3.45±0.44	3.76±0.44	4.23±0.90	4.63±1.20	**<0.001**	0.117	0.835
E’ septal (cm/s)[*]	8.72±11.8	6.17±0.44	4.40±0.60	4.94±0.60	**<0.001**	0.207	0.158
E’ lateral (cm/s)	5.33±0.44	5.47±0.44	4.07±0.90	4.61±0.90	**<0.001**	0.145	0.389

Data are expressed as mean ± standard deviation. n: numbers of animals included in each experimental group. SM: sham animals with maltodextrin intake; SOJ: sham animals with orange juice intake; IM: infarcted animals with maltodextrin intake; IOJ: infarcted animals with orange juice intake. BW: body weight; PWT: LV posterior wall thickness; ISWT: interventricle septum wall thickness; LA: left atrium diameter; HR: heart rate; FS: endocardial fractional shortening; EF: ejection fraction; Tei index: myocardial performance index; E/A: e wave and A wave ratio; EDT: E wave deceleration time; $IRT/RR^{0.5}$: isovolumetric relaxation time adjusted by heart rate; S′: systolic annular mitral velocity septal and lateral; A′ and E′ diastolic annular mitral velocity septal and lateral (E′: early and A′: late). pI: *p* value of infarct effect. pOJ: *p* value of orange juice intake effect. pIxOJ: *p* value of interaction. Bold numbers represent the significant effects that were considered. [a]: IM≠IOJ; [b]: SM≠SOJ; [A]: SM≠IM e [B:] SOJ≠IOJ.

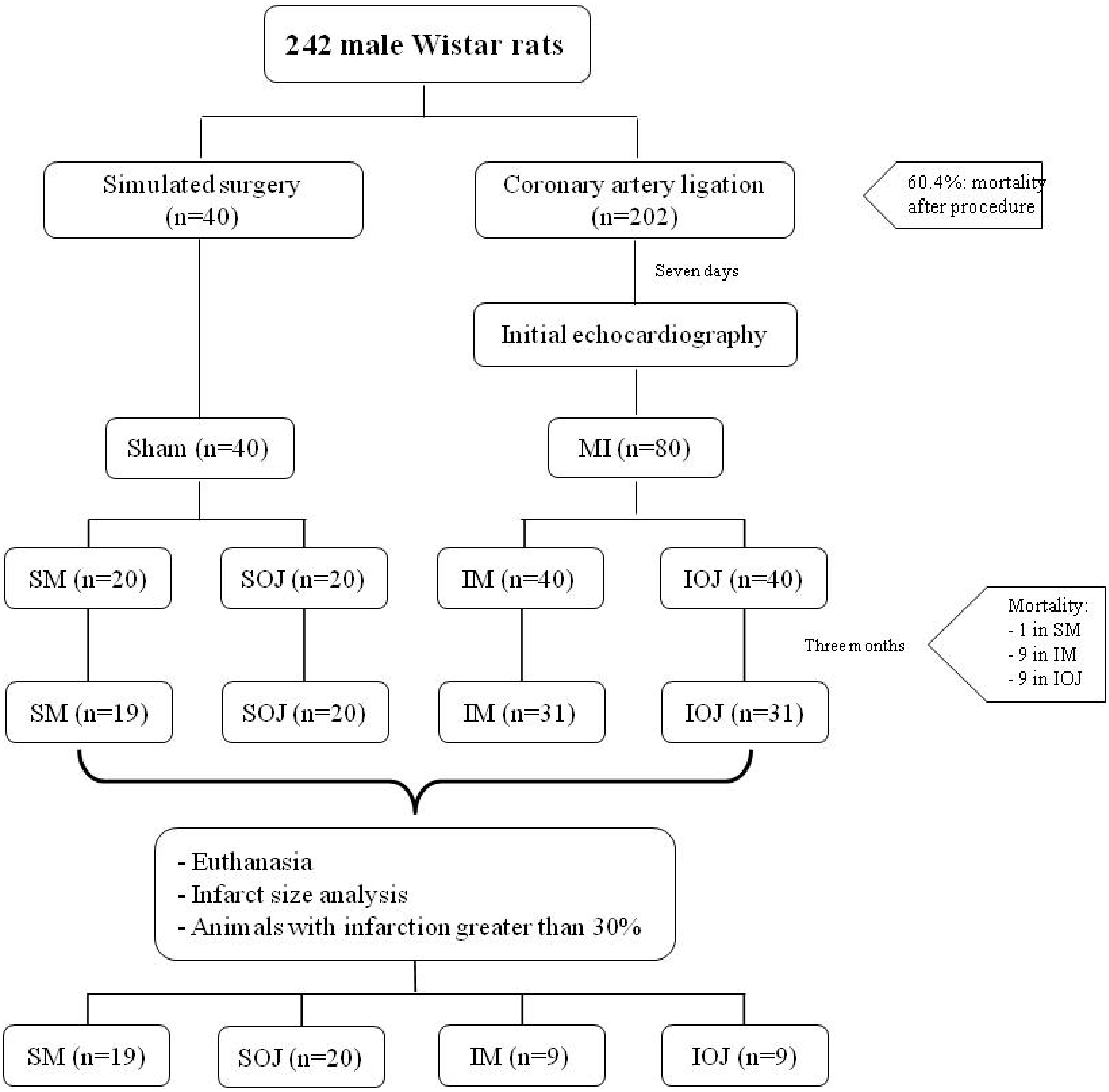

Figure 1. Study design, experimental groups, number of animals, mortality, and infarct size.

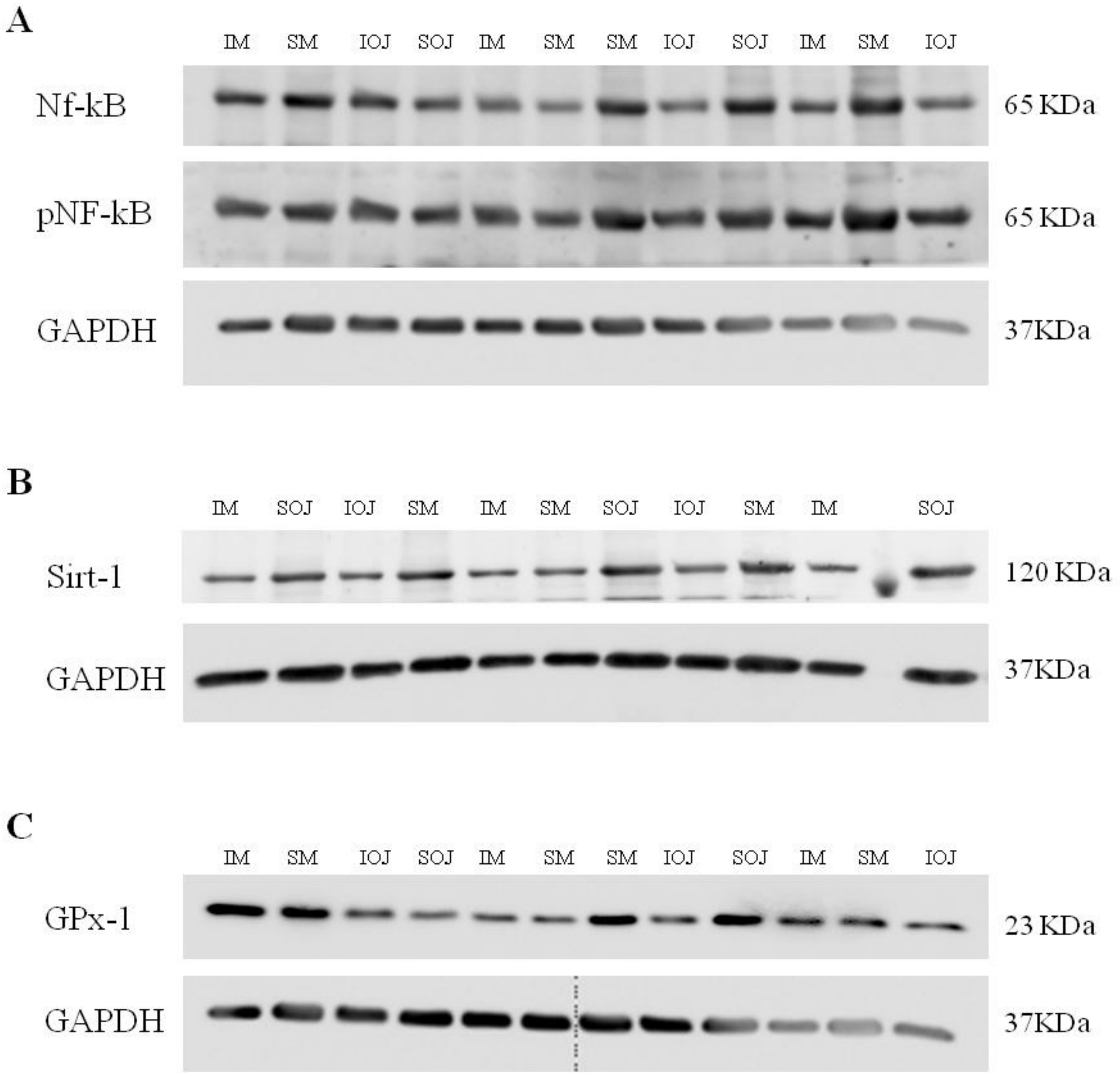

Figure 2. Nf-kB, pNF-kB, Sirt-1 and GPx-1 representative Western blot in sham and infarcted rats.

(A) Nf-kB and pNF-kB representative Western blot. (B) Sirt-1 representative Western blot. (C) GPX-1 representative Western blot. NF-kB: total nuclear factor-kB; pNF-kB:phosphorylated nuclear factor-kB; Sirt-1: sirtuin-1; GPx-1: glutathione peroxidase-1.
