# Supplementary material for: Influência do Consumo de Suco de Laranja (Citrus Sinensis) na Remodelação Cardíaca de Ratos Submetidos a Infarto do Miocárdio
Source: Arq Bras Cardiol. 2021 Jun 8;116(6):1127–36. [Article in Portuguese] doi: 10.36660/abc.20190397 (PMC8288524; doi:10.36660/abc.20190397)
Supplement: Translation

# Translation: Influence of Consumption of Orange Juice (Citrus Sinensis) on Cardiac Remodeling of Rats Submitted to Myocardial Infarction

**Authors:** Bruna C. Oliveira, Priscila P. Santos, Amanda M. Figueiredo, Bruna P. M. Rafacho, Larissa Ishikawa, Silméia G. Zanati, Ana A. H. Fernandes, Paula S. Azevedo, Bertha F. Polegato, Leonardo A. M. Zornoff, Marcos F. Minicucci, Sergio A. R. Paiva

**Affiliations:** 1 Brazil Universidade Estadual Paulista Júlio de Mesquita Filho Campus de Botucatu - Faculdade de Medicina de Botucatu, Botucatu, SP - Brazil; 2 Brazil Instituto de Biociências Campus de Botucatu (UNESP), Botucatu, SP - Brazil; 3 Brazil Food Research Center FoRC, São Paulo, SP - Brazil

**Keywords:** Myocardial Infarction, Fruits Juices (orange), Polyphenols, Ventricular Remodeling, Anti-Inflammatory Agents, Antioxidants

## Abstract

**Background:**

Orange juice (OJ) is rich in polyphenols with anti-inflammatory and antioxidant properties. After myocardial infarction (MI), complex changes occur in cardiac structure and function, which is known as cardiac remodeling (CR). Oxidative stress and inflammation can modulate this process. We hypothesized that the consumption of OJ attenuates the CR after MI.

**Objectives:**

To evaluate the influence of OJ on CR after MI by analysis of functional, morphological, oxidative stress, inflammation, and energy metabolism variables.

**Methods:**

A total of 242 male rats weighing 200-250 g were submitted to a surgical procedure (coronary artery ligation or simulated surgery). Seven days after surgery, survivors were assigned to one of the four groups 1) SM, sham animals with water and maltodextrin (n= 20); 2) SOJ, sham animals with OJ (n= 20); 3) IM, infarcted animals with water and maltodextrin (n= 40); and 4) IOJ, infarcted animals with OJ (n = 40). Statistical analysis was performed by the two-way ANOVA supplemented by Holm-Sidak. Results are presented as mean ± standard deviation, the level of significance adopted was 5%.

**Results:**

After 3 months, MI led to left ventricular (LV) hypertrophy, with systolic and diastolic dysfunction, and increased oxidative stress and inflammatory mediators. OJ intake reduced LV cavity and improved systolic and diastolic function. The OJ animals presented lower activity of glutathione peroxidase and higher expression of heme-oxygenase-1 (HO-1).

**Conclusion:**

OJ attenuated CR in infarcted rats and HO-1 may be play an important role in this process.

## Introduction

The name *polyphenols* , or *phenolic compounds* , refers to a large group of molecules found in leaf vegetables, fruits, cereals, tea, coffee, cocoa, wine, soy, and fruit juice.^1^ These compounds have been studied because of their potential biological effect in prevention and treatment of different diseases.^2 , 3^

In a review of the literature, Hyson showed that fruit juices, defined as pure juice or 100% fruit juice, retained most of nutrients and phytochemicals of the whole fruit and therefore may have an important potential to benefit and protect human health.^4^ Orange juice (OJ) is a source of phenolic compounds in the form of different flavonoids. The main flavonoid of interest is hesperidin and its hydrolyzed form, hesperetin.^5^ The research interest in the properties of OJ has increased because of its anti-inflammatory and antioxidant action in chronic diseases.^6^

For example, in myocardial injury, antioxidant supplements may have beneficial effects in cardiac remodeling (CR). In studies using myocardial infarction (MI) model, bioactive compounds present in rosemary, tomato, green tea, and antioxidants such as ascorbic acid, quercetin, alpha-tocopherol, and vitamin A showed protection against CR.^1 , 3 , 7 - 10^

Ischemic heart disease, including MI, is a leading cause of heart failure and death worldwide. After MI, complex changes in the left ventricle can cause changes in cardiac size, mass, geometry, and function.^11 , 12^ These changes are defined as CR and can lead to heart failure and increased mortality.^13^ Many factors may be involved in CR, such as oxidative stress, inflammation, fibrosis, and apoptosis.^14 , 15^

In MI, ischemia initiates the generation of reactive oxygen species (ROS). ROS directly damage cell membranes, activate the inflammatory response, and lead to cell death. They can also act as transduction signals, stimulating nuclear factor κB (NF-κB), which, in turn, stimulates proinflammatory cytokines synthesis.^14 - 16^ In addition, the Kelch-like ECH-associated protein 1/nuclear factor erythroid 2-related factor 2 (KEAP-1/Nrf2) system could be activated during cellular oxidative stress and play a critical role in redox homeostasis. This is a universal mechanism that acts in Nrf2 target genes called antioxidant response elements. Glutathione peroxidase (GSH-Px) and heme-oxygenase-1 (HO-1) are examples of proteins regulated by this system.^17^

Therapeutic strategies to attenuate CR after MI have been extensively studied.^18 , 19^ Aldosterone blockers, angiotensin-converting enzyme inhibitors, and beta-blockers are some of these strategies.^20^ In this context, bioactive compounds of natural products, with cardioprotective properties such as flavonoids, can be an important adjuvant in the treatment of MI. On the other hand, studies show that a focus on food and dietary patterns instead of individual nutrients or phytochemicals is better for cardiometabolic health.^21^ Thus, the aim of this study was to evaluate the influence of OJ intake on CR after MI.

## Materials and Methods

### Experimental protocol

All experiments and procedures were performed in accordance with the National Institutes of Health’s (NIH) Guide for the Care and Use of Laboratory Animals and were approved by the Ethics Committee for Animal Experimentation of the Botucatu Medical School, UNESP, São Paulo, Brazil (1126/2015). A total of 242 male Wistar rats weighing 200 to 250 g were used in this study. MI was induced by coronary artery ligation, as previously described.^22 , 23^

After surgery, the animals were placed in boxes with six animals each. Seven days after surgical procedure, the first echocardiographic study was performed to evaluate the efficacy of the surgical procedure.^24^ Then, the animals were randomly placed in boxes with two animals each, to receive either OJ or a maltodextrin (M) solution. The groups were 1) SM, sham animals that received M solution (n = 20); 2) SOJ, sham animals that received OJ (n = 20); 3) IM, infarcted animals that received M solution intake (n = 40); and 4) IOJ, infarcted animals that received OJ (n = 40). The sample size used was based on other studies from our laboratory.^3 , 8 , 25^ The number of rats in the infarcted groups was higher, since the expected mortality in these animals during the experimental period is around 50%. In addition, only rats with a left ventricular (LV) infarcted area greater than 30% were included.^24^

Food was supplied *ad libitum* . The animals were treated for 3 months, and mortality was observed in this period ( Figure 1 of the supplementary data). The rats were housed in a temperature-controlled room (22 ± 2°C) with a 12-h light/12-h dark cycle.

**Figure 1:** – Nuclear factor erythroid 2-related factor 2 (Nrf2) and heme-oxygenase-1 (HO-1) expression in sham and infarcted rats by Western blot. Bar chart showing the expression of Nrf2 and HO-1 proteins in each group (A) Nrf2 expression and representative Western blot; sample size: 8 in each group. (B) HO-1 expression and representative Western blot; sample size: SM = 5; SOJ = 6; IM = 5; IOJ = 5. GAPDH glyceraldehyde-3-phosphate dehydrogenase. Data are expressed as mean ± SD. p(I): p value between non-infarcted animals vs. infarcted animals; p(OJ): p value between animals that received maltodextrin vs. animals that received orange juice; p(IxOJ): p-value for the interaction between the factors of infarction and orange juice intake.

### Coronary artery ligation

MI was conducted by coronary artery ligation, as previously described.^22 , 23^ In brief, the rats were anesthetized with ketamine (70 mg/Kg) and xylazine (1 mg/Kg), and after a left thoracotomy, the heart was exteriorized. The left atrium was retracted to facilitate the ligation of the left coronary artery with 5-0 mononylon between the pulmonary outflow tract and the left atrium. The heart was then replaced in the thorax, the lungs were inflated by positive pressure, and the thoracotomy was closed. A sham group, in which animals were submitted to surgery but without coronary occlusion, was also created. After anesthetic effect, the rats were medicated orally with metamizole sodium (30 mg / kg Dipirona®, Biovet, Vargem Grande Paulista, Sao Paulo, Brazil).

### Orange juice

Supplemented groups (SOJ and IOJ) received OJ *ad libitum* . Control groups (SM and IM) received a solution of water and M at a concentration of 100 g/L. The M solution was given to control animals to provide the same amount of carbohydrates as the OJ. Treatment began seven days after surgery. The OJ and M solutions were changed every 24 hours, and intake was monitored daily. Nutritional composition of the OJ is shown in supplementary data.

### Echocardiographic study

After three months, all rats were weighed and evaluated by transthoracic echocardiography.^26 , 27^ For the echocardiographic study, the rats were anaesthetized with intramuscular injection of ketamine (50 mg/kg) and xylazine (1 mg/kg) solution. All measurements were made by the same observer, according to the leading-edge method recommended by the American Society of Echocardiography/European Association of Echocardiography.^28^ Echocardiography was performed with the General Electric Vivid S6 System (GE Medical Systems, Tirat Carmel, Israel) equipped with a 5- to 12-MHz phased array transducer.

After echocardiography, the animals were euthanized with a large dose of pentobarbital, and their hearts were removed. The left ventricle was isolated and LV samples were immediately frozen and stored at –80°C. One transverse section of the LV was separated and fixed in 10% buffered formalin and was then embedded in paraffin for histological study.

### Morphometric analysis

Five-micrometer-thick sections were stained with hematoxylin and eosin for calculations of infarction size as previously described. All animals were included in the morphometric analysis. After infarction size calculation, infarcted animals with less than 30% of LV infarcted area were excluded from analysis. All images were collected with a video camera attached to Leica microscope; the images were analyzed with the Image-Pro Plus 3.0 software program (Media Cybernetics, Silver Spring, MD).

### Cardiac lipid hydroperoxide, antioxidant enzyme activity, and cardiac energy metabolism

LV samples (100 mg) were used to determine total protein and lipid hydroperoxide (LH) concentrations, and activity of the following antioxidant enzymes – GPx (E.C.1.11.1.9), superoxide dismutase (SOD, E.C.1.15.1.1), and catalase (E.C.1.11.1.6). Cardiac energy metabolism was assessed by 3-hydroxyacyl coenzyme-A dehydrogenase (OHADH; E.C.1.1.1.35.), phosphofructokinase (PFK; E.C.2.7.1.11), lactate dehydrogenase (LDH; E.C.1.1.1.27), pyruvate dehydrogenase (E.C.1.2.4.1), citrate synthase (CS; E.C.4.1.3.7.), and adenosine triphosphate (ATP) synthase (EC 3.6.3.14) activities.^3 , 9^ The enzyme activity assays were performed at 25°C with a microplate reader (µQuant-MQX 200-EONC with Gen5 2.0 software connected to a computer system control; Bio-Tec Instruments, VT, USA). All the reagents were obtained from Sigma (Sigma-Aldrich, St. Louis, MO, USA).

### Inflammatory mediators

Interferon-γ (IFN-γ) and interleukin-10 (IL-10) concentrations in LV samples were determined by ELISA according to the manufacturer’s instructions (R&D Systems, Minneapolis, MN).

### Western blot

Western blot was performed to analyze protein expression of GPx-1 (ab 22604 - Abcam Inc, Cambridge), HO-1 (ab13248 - Abcam Inc, Cambridge), total and phosphorylated NF-kB (NF-kB- sc 8008 and sc 3302- Santa Cruz Biotechnology, Inc, Europe), and sirtuin-1 (Sirt-1- sc 15404-Santa Cruz Biotechnology, Inc, Europe), in total cellular extract. To determine nuclear erythroid factor 2 (Nrf-2-sc 722-Santa Cruz Biotechnology Inc, Europe), LV samples were extracted with nuclear extraction buffer.^9^ Samples were separated on a 10% sodium dodecyl sulfate–polyacrylamide gel, and the proteins were transferred to a nitrocellulose membrane. The membrane was blocked with 5% nonfat dry milk and then incubated with primary and secondary antibodies. Glyceraldehyde-3-phosphate dehydrogenase GAPDH (sc 32233, Santa Cruz Biotechnology, Inc., Europe) was used for normalization of all proteins.

### Statistical analysis

The normality of the data was verified by Kolmogorov–Smirnov statistical test. Data are presented as the mean ± standard deviation (SD). Variables with normal distributions were analyzed by 2-factor analysis of variance, which gives three *p* values: 1) factor 1, presence of MI (I); 2) factor 2, OJ intake (OJ); and 3) interaction between factors I and OJ. The 2-factor analysis of variance requires an assumption of normality. If a measurement variable does not fit a normal distribution, data transformation was performed. The Student’s unpaired *t* -test was used to analyze the initial echocardiogram; the χ^2^test was used to evaluate mortality, and Student’s unpaired t-test used to evaluate the infarct size in infarcted animals. Differences were considered statistically significant if *p* <0.05. Statistical analyses were performed using SigmaPlot for Windows 12.0 (Systat Software Inc., San Jose, CA).

## Results

The initial echocardiogram showed that animals of both infarcted groups did not present differences in the systolic and diastolic area or in the infarct size ( Table 1 of Supplementary material).

**Table 1:** – Infarction size, final echocardiogram, and morphometric analysis.

Variable	SM (n = 19)	SOJ (n = 20)	IM (n = 9)	IOJ (n = 9)	p (I)	p (OJ)	p (I×OJ)
BW (g)	454±47.9	480±56.3	443±66.6	462±25.9	0.338	0.135	0.852
HR (bpm)	290±30.9	296±34.9	268±26.7	324±30.0^aB^	0.756	0.001	0.009
LA/Ao	1.32±0.09	1.21±0.09^b^	1.78±0.21^A^	1.42±0.18^Ba^	<0.001	<0.001	0.003
LVEDD/BW (mm/kg)	15.9±1.31	15.0±1.79	22.1±2.70	20.2±2.10	<0.001	0.019	0.398
LVESD/BW (mm/kg)	6.67±0.87	6.02±0.89	15.1±2.70	12.8±2.70	<0.001	0.007	0.556
LVMI (g/kg)	1.63±0.22	1.56±0.27	2.62±0.60	2.36±0.36	<0.001	0.124	0.640
FAC (%)	67.3±8.28	67.4±8.27	34.5±8.28	36.6±8.28	<0.001	0.661	0.707
E wave (ms)	79.7±11.3	81.4±7.16	89.1±10.8	80.7±19.2	0.204	0.325	0.139
A wave (ms)	49.9±8.28	52.6±7.16	42.5±16.5	62.7±24.9^a^	0.730	0.004	0.025
S´ mean (cm/s)	5.78±0.04	5.82±0.31	4.60±0.30	5.01±0.3	<0.001	0.028	0.078
E´ mean (cm/s)	5.62±0.44	5.82±0.45	4.23±0.60	4.77±0.60	<0.001	0.054	0.357
A´ mean (cm/s)	3.67±0.44	4.02±0.45	4.54±0.90	5.55±1.20	0.058	0.058	0.28
E/E´ ratio	13.7±3.49	14.1±1.79	21.3±3.30^A^	16.9±2.40^Ba^	<0.001	0.002	0.003
LVW/BW (mg/g)	1.85±0.13	1.93±0.27	2.13±0.42	2.10±0.15	0.005	0.550	0.822
RVW/BW (mg/g)	0.46±0.09	0.43±0.05	0.65±0.24	0.59±0.27	<0.001	0.294	0.776

*Data are expressed as mean ± standard deviation. n: numbers of animals included in each experimental group. SM: sham animals that received maltodextrin; SOJ: sham animals that received orange juice; IM: infarcted animals that received maltodextrin; IOJ: infarcted animals that received orange juice. BW: body weight; HR: heart rate; LA: left atrial diameter; Ao: aorta diameter; LVEDD: left ventricular end-diastolic diameter; LVESD: left ventricular end-systolic diameter; LVMI: left ventricular mass index (left ventricular mass/BW); FAC: fractional area change; E wave: early diastolic mitral inflow velocity; A wave: late diastolic mitral inflow velocity; S´ mean: mean of systolic annular mitral velocity septal and lateral; A´ and E´ mean: mean of diastolic annular mitral velocity septal and lateral (E´: early and A: late); LVW: left ventricular weight; RVW: right ventricular weight. pI: p value for the effect of infarction. pOJ: p value of the effect of orange juice intake. pIxOJ: p value of interaction. Bold numbers represent statistically significant effects;^a^: IM≠IOJ;^b^: SM≠SOJ;^A^: SM≠IM e^B:^SOJ≠IOJ.*

During the 3-month experimental period, mortality was 5.0% in the SM (1 rat died), 0% in the SOJ, 22.5% in the IM (9 rats died), and 22.5% in the IOJ group (9 rats died). When all groups were analyzed, a difference in mortality was observed between the groups (p=0.04). However, mortality was not different between the infarcted groups (p=0.836). After the period of OJ intake, euthanasia of surviving animals was performed. Then, histological analysis of the left ventricle of infarcted animals was performed to verify the infarction size ( Figure 1 of the Supplementary material). These animals did not present a difference in infarct size (IM= 40.1±7.41%; IOJ=38.1±5.76%; p= 0.528). The final body weight (BW) was not different among the groups ( Table 1 ).

### Effect of MI in rat hearts

MI led to adverse CR. Regarding morphological data, MI led to higher values of LV diastolic diameter/BW, LV systolic diameter/BW, left atrial diameter/aorta, LV mass index (LVMI), LV weight/BW, and right ventricular weight/BW ( Table 1 ), LV posterior wall thickness/BW, interventricular septum wall thickness/BW, and left atrial diameter/BW ( Table 1 of Supplementary material). These changes characterize the enlargement of the left cavities and LV hypertrophy. MI impaired cardiac systolic function, as shown by the lower values of fractional area change (FAC), S´ mean ( Table 1 ), endocardial fractional shortening, and ejection fraction ( Table 2 of Supplementary material). Cardiac diastolic function was also impaired, as shown by decreased mean E´ ( Table 1 ), E wave deceleration time, E´ lateral, and E´ septal ( Table 2 of Supplementary material) and increased A wave, A´ mean; E/E´ ratio ( Table 1 ), Tei index, E/A ratio, isovolumetric relaxation time adjusted by heart rate, A´ lateral, and A´ septal.

**Table 2:** – Oxidative stress and inflammatory markers

Variable	SM (n = 8)	SOJ (n = 8)	IM (n = 4)	IOJ (n = 4)	p (I)	p (OJ)	p (I×OJ)
LH (nmol/g)	209±28.3	215±28.3	256±28.0	277±26.0	<0.001	0.293	0.573
Catalase (µmol/g)	64.4±6.79	61.8±7.07	55.4±7.00	58.1±7.60	0.056	0.993	0.404
SOD (nmol/mg)	12.9±2.83	13.5±2.83	18.7±1.80	17.1±1.40	<0.001	0.639	0.355
GSH-px activity (nmol/mg)	62.5±6.51	50.2±7.07	58.0±7.00	45.3±6.00	0.134	<0.001	0.951
GSH-px expression (arbitrary unit)	7.56±7.39	7.62±10.8	6.63±5.82	6.37±3.78	0.600	0.649	0.546
IL-10 (pg/mg)	23.9±7.92	39.6±13.0^b^	42.0±9.60^A^	34.1±7.60	0.135	0.352	0.008
IFN-γ (pg/mg)	7.1±2.26	13.2±3.96^b^	17.7±6.80^A^	11.2±2.00^a^	0.037	0.928	0.003
NF-κB (arbitrary unit)	7.03±11.0	4.61±4.98	3.40±3.12	6.80±4.00	0.942	0.330	0.266
p NF-κB (arbitrary unit)	5.40±10.2	3.18±2.40	3.28±3.12	5.22±3.34	0.587	0.084	0.980
Sirt 1 (arbitrary unit)	1.81±0.82	2.34±1.41	2.42±1.44	1.65±0.56	0.925	0.813	0.203

*Data are expressed as mean ± standard deviation. n: numbers of animals included in each experimental group. SM: sham animals that received maltodextrin; SOJ: sham animals that received orange juice; IM: infarcted animals that received maltodextrin; IOJ: infarcted animals that received orange juice. LH: lipid hydroperoxide; SOD: superoxide dismutase; GSH-px: glutathione peroxidase; IL-10: interleukin-10; INF-γ: interferon- γ; NF-κB: nuclear factor–κB total and phosphorylated (p NF-κB); Sirt 1: sirtuin 1. pI: p value for the effect of infarction. pOJ: p value of orange juice intake effect. pIxOJ: p value of interaction.Bold numbers represent statistically significant effects.* ^*a*^ *: IM≠IOJ;* ^*b*^ *: SM≠SOJ;* ^*A*^ *: SM≠IM e* ^*B:*^ *SOJ≠IOJ.*

MI also increased oxidative stress, as presented with higher LH and SOD activity ( Table 2 ), lower expression of HO-1 (Figure 1A), and of Nrf2 (Figure 1B). The inflammatory mediators IL-10 and INF-γ were higher in MI ( Table 2 ), and there was no difference in NF-κB and Sirt-1 between infarcted and noninfarcted animals ( Table 2 and Figure 2 of Supplementary material). A greater oxidation of carbohydrates than fatty acids and impaired energy metabolism were observed, as shown by higher activity of LDH and PFK and lower activity of OHADH, CS, and ATP synthase. No difference was observed for activity of pyruvate dehydrogenase ( Table 3 ).

**Figure 2:** – Scheme illustrating the main findings of this study.

**Table 3:** – Enzymes involved in cardiac energy metabolism

Variable	SM (n = 8)	SOJ (n = 8)	IM (n = 4)	IOJ (n = 4)	p (I)	p (OJ)	p (I×OJ)
PFK (nmol/g)	128±17.0	112±24.6	139±26.0	178±26.0^aB^	<0.001	0.257	0.011
LDH (nmol/mg)	88.5±18.7	82.6±18.1	111±18.6	134±16.	<0.001	0.320	0.092
PDH (nmol/g)	344±53.7	385±31.1	345±100	341±42.0	0.386	0.461	0.376
OHADH (nmol/mg)	33.2±6.22	33.9±6.22	23.8±8.40	24.5±2.80	0.003	0.798	0.992
CS (nmol/mg)	50.0±6.22	49.4±6.51	34.5±7.40	40.5±4.80	<0.001	0.350	0.254
ATP synthase (nmol/mg)	21.0±3.11	27.8±5.37	11.4±1.20	15.9±4.40	<0.001	0.005	0.532

*Data are expressed as mean ± standard deviation. n: numbers of animals included in each experimental group. SM: sham animals that received maltodextrin; SOJ: sham animals that received orange juice; IM: infarcted animals that received maltodextrin; IOJ: infarcted animals that received orange juice. PFK: phosphofructokinase; LDH: lactate dehydrogenase; PDH: pyruvate dehydrogenase; OHADH: 3-hydroxyacyl coenzyme-A dehydrogenase; CS: citrate synthase; ATP: adenosine triphosphate. pI: p value for the effect of infarction. pOJ: p value of orange juice intake effect. pIxOJ: p value of interaction. Bold numbers represent statistically significant effects.^a^: IM≠IOJ;^b^: SM≠SOJ;^A^: SM≠IM e^B:^SOJ≠IOJ.*

### Effect of OJ intake on rat hearts

OJ intake reduced the LV cavity, with lower values of LV end-diastolic diameter (LVDD) and LV end-systolic diameter (LVSD); improved systolic function, with higher values of S´ mean; and improved diastolic function, with lower left atrial diameter adjusted for aorta diameter ( Table 1 )^29^ after MI. No differences were observed for other echocardiographic variables ( Table 1 ). The other function variables were not valued because of the higher heart rate^30^ in the IOJ group (presented in Supplementary material Table 2 ).

In addition, the animals that consumed OJ presented lower activity of GSH-Px ( Table 2 ). No differences were observed for LH, SOD activity, catalase activity, or GSH-Px expression ( Table 2 and Figure 2 of the Supplementary material).

Regarding the inflammatory mediators, the SOJ group showed higher IL-10 and INF-γ than the SM group ( Table 2 ). Also, the IOJ group had lower INF-γ values than the IM group ( Table 2 ). No differences were observed for NF-κB or Sirt-1 between animals that consumed or did not consume OJ ( Table 2 and Figure 2 of the Supplementary material).

An improvement in the use of substrate occurred in the animals that consumed OJ. We observed higher values in PFK activity in the IOJ group compared with the IM group and higher activity of ATP synthase in animals that consumed OJ. No difference was observed in the activity of other energy metabolism enzymes between the groups ( Table 3 ).

Interestingly, the animals with OJ intake had a higher expression of HO-1 (Figure 1A), although they did not present a difference in Nrf2 expression (Figure 1B).

## Discussion

In the present study, MI induced by coronary artery ligation in rats resulted in LV hypertrophy and diastolic dysfunction, which was compatible with changes observed in chronic infarction.^11^ Our data also showed increased oxidative stress and inflammatory markers as well as alterations in energy metabolism, with impairment of fatty acid β-oxidation. These alterations characterize the CR process.^31 - 33^ We also observe a decrease in Nrf2 and HO-1 expression. In the chronic phase of MI, the Nrf2 pathway may be diminished by abnormal expression of the Nrf2 target gene, affecting the maintenance of redox homeostasis via enzymes mediated by antioxidant response elements.^34^ In a previous study conducted in our laboratory with the MI model, lower expression of Nrf2 and HO-1 was observed.^3^ These findings suggest either a lower expression or greater catabolism of the Nrf2 protein, thus leading to the lower synthesis of HO-1.

In the present study, OJ intake resulted in attenuation of CR in infarcted animals. This attenuation can be observed in the decrease of LV cavity (LVDD and LVSD) and in the improvement of systolic function, characterized by the increase in the S´ mean,^35^ and diastolic function (lower left atrial diameter). In the study by Yu et al.,^36^ infarcted rats, by left coronary ligation, treated with hesperidin for four weeks had lower LVDD and LVSD and improved systolic function than infarcted animals. These data are similar to ours and may show the effect of hesperidin of OJ on the CR process. In other study, another phenolic compound, hesperetin, also had an effect on the heart. In pressure-overload model, Deng et al.^37^ found lower values of LVDD and LVSD at eight weeks of hesperetin administration.

MI leads to an imbalance between the production of ROS and antioxidant defenses, leading to oxidative stress. After ischemia, some ROSs damage cell membranes, initiating the process of lipid peroxidation.^38^ For example, Bagatini et al.^39^ described an increase in lipid peroxidation in patients with MI. Our data also show a higher concentrations of lipid hydroperoxides in infarcted animals compared to non-infarcted animals. The SOD enzyme is the organism’s first defense against ROS.^40^ Our data show that infarcted animals compared to non-infarcted animals showed greater activity of the SOD enzyme, as previously described.^3 , 25^ Regarding OJ intake, we observed that the animals that consumed OJ presented lower GSH-Px activity. A similar result was shown by Selvaraj and Pugalendi^41^ in myocardial ischemia model induced by isoproterenol: rats that received hesperidin presented lower activity of the antioxidant enzymes, among them GSH-Px.^41^

In relation to energy metabolism, the heart, like other organs, can adapt and use the best energy substrate in each situation. PFK acts in glycolysis regulation, and catalyzes the phosphorylation of glucose in fructose-6-phosphate and subsequently in fructose 1,6-bisphosphate.^42^ PFK is activated when ATP concentrations become low and is inhibited when cells have sufficient supply of ATP and other substrates such as fatty acids.^42^ Our data showed higher values of PFK in infarcted animals with OJ intake. These data show that increased PFK activity may lead to regulation of the glycolytic pathway, by providing more substrate for energy production. Another important finding that indicates a greater use of substrate is the higher ATP synthase activity in the animals that received OJ.

In addition to oxidative stress and metabolic changes, we found that the infarcted animals that received OJ presented lower values of IFN-γ. Since a chronic phase of inflammation is related to an increased production of IFN-γ,^43^ our findings suggest more advanced phase towards the resolution of the inflammatory process. An interesting result is that sham animals who consumed OJ demonstrated an immunomodulatory effect, as shown by higher values of IL-10 and INF-γ. Similar to our findings in animals in the sham group, which had no cardiac injury, studies in healthy, middle-aged humans showed that OJ altered leukocyte gene expression to an anti-inflammatory and anti-atherogenic profile^44^ and provided an early protection of mononuclear blood cell against oxidative DNA damage.^45^ In addition, OJ intake with the high-carbohydrate meal prevented meal-induced oxidative and inflammatory stress.^46^

Another intriguing finding in our study was the higher values of HO-1 in animals that consumed OJ. Lin et al.^47^ in 2005 also showed that hesperetin induced protein expression of HO-1.^47^ The HO-1 enzyme plays important role in cell homeostasis because of its catabolic action on the heme group of hemoproteins, generating by-products such as iron, biliverdin, and carbon monoxide. Through these by-products, HO-1 exerts anti-inflammatory, antioxidant, and antiapoptotic action.^48 , 49^ In addition to this classical function, HO-1 participates in cell signaling as amplifier of inductors (heme, oxidants, cytokines, hemodynamic forces, growth factors, hypoxia, and hormones) of transcription factors.^48^

Wang et al.^50^ showed that HO-1 is important for heart homeostasis by protecting it from ischemia and reperfusion-induced lesions and oxidative damage.^50^ In another study, hemin administration in infarcted mice induced HO-1 activation, which caused a change in infarct macrophages toward a M2 anti-inflammatory phenotype, reduction of infarct scar expansion, and improvement of cardiac function.^51^ Thus, increased HO-1 may also play an important role in CR attenuation by OJ ( Figure 2 ). In addition, this increase was independent of the Nrf2 pathway, since OJ did not lead to alterations in the expression of this protein. Similar to our findings, Wang et al.^52^ found that isoliquiritin and isoliquiritigenin, flavonoids derived from liquorice, induced HO-1 expression independent of Nrf2 expression.^52^ The HO-1 expression can be induced by different pathway and may vary according to the model and treatment used.^47^

### Limitations

The OJ used in the study was a commercial, ready-to-eat, pasteurized juice, free of preservatives and sugar. The choice of ready juice was to ensure its standardization. However, the use of other types of juices, made with other types of oranges, could possibly show different responses.

## Conclusion

OJ attenuated CR after MI, with decreased LV diameter as well as improvement in systolic and diastolic function; HO-1 may play an important role in this process.

## * Supplemental Materials

For additional information, please click here.
